# CUSHAW3: Sensitive and Accurate Base-Space and Color-Space Short-Read Alignment with Hybrid Seeding

**DOI:** 10.1371/journal.pone.0086869

**Published:** 2014-01-22

**Authors:** Yongchao Liu, Bernt Popp, Bertil Schmidt

**Affiliations:** 1 Institut für Informatik, Johannes Gutenberg Universität Mainz, Mainz, Germany; 2 Institute of Human Genetics, University of Erlangen-Nuremberg, Erlangen, Germany; Harvard School of Public Health, United States of America

## Abstract

The majority of next-generation sequencing short-reads can be properly aligned by leading aligners at high speed. However, the alignment quality can still be further improved, since usually not all reads can be correctly aligned to large genomes, such as the human genome, even for simulated data. Moreover, even slight improvements in this area are important but challenging, and usually require significantly more computational endeavor. In this paper, we present CUSHAW3, an open-source parallelized, sensitive and accurate short-read aligner for both base-space and color-space sequences. In this aligner, we have investigated a hybrid seeding approach to improve alignment quality, which incorporates three different seed types, i.e. maximal exact match seeds, exact-match *k*-mer seeds and variable-length seeds, into the alignment pipeline. Furthermore, three techniques: weighted seed-pairing heuristic, paired-end alignment pair ranking and read mate rescuing have been conceived to facilitate accurate paired-end alignment. For base-space alignment, we have compared CUSHAW3 to Novoalign, CUSHAW2, BWA-MEM, Bowtie2 and GEM, by aligning both simulated and real reads to the human genome. The results show that CUSHAW3 consistently outperforms CUSHAW2, BWA-MEM, Bowtie2 and GEM in terms of single-end and paired-end alignment. Furthermore, our aligner has demonstrated better paired-end alignment performance than Novoalign for short-reads with high error rates. For color-space alignment, CUSHAW3 is consistently one of the best aligners compared to SHRiMP2 and BFAST. The source code of CUSHAW3 and all simulated data are available at http://cushaw3.sourceforge.net.

## Introduction

The emergence and rapid progress of next-generation sequencing (NGS) technologies has driven a substantial amount of research efforts into the development of short-read alignment algorithms. To date, a variety of short-read aligners have been developed, which can be further classified into two generations in terms of functionality. The first-generation aligners are usually designed and optimized for very short reads (typically ≤100 bps). These aligners usually postulate that the short-reads have very small deviations from the genome, and thus typically only allow mismatches. Even though some aligners provide support for gaps, the maximum allowable number of gaps is also quite limited (typically one gap) for the sake of speed. Example first-generation aligners include RMAP [1], MAQ [2], BFAST [3], Bowtie [4], BWA [5], CUSHAW [6] and SOAP3 [7].

With the progress of NGS, the maximum or average read lengths are steadily increasing beyond 100 for Illumina sequencing, which is most widely used. However, these longer short-reads usually come at the expense of higher sequencing error rates. On the other hand, these reads are prone to have more true insertions or deletions (indels) to the genome. These new features make the first-generation aligners become inefficient to align such longer reads in terms of alignment quality, speed or even both, and thus motivate the development of second-generation aligners that allow for fully gapped alignments with more mismatches and indels supported.

Several second-generation aligners have been developed recently, including BWA-SW [8], GASSST [9], Bowtie2 [10], CUSHAW2 [11], GEM [12], SeqAlto [13], SOAP3-dp [14] and BWA-MEM [15]. All these aligners are designed based on the seed-and-extend paradigm. In this paradigm, a read is aligned by first identifying seeds, i.e. short ungapped/gapped alignments, on the genome and then extending the alignment to the rest of the read using dynamic programming. Constraints and filtrations are often exerted on alignment extensions to further reduce search space. Different seeding polices may be employed by different aligners. BWA-SW employs variable-length gapped seeds, and Bowtie2 extracts fixed-length ungapped seeds (inexact matches). Both GASSST and SeqAlto employ fixed-length exact-match *k*-mer (a *k*-mer is a substring of *k* bases) seeds, while CUSHAW2 and BWA-MEM respectively identifies variable-length maximal exact match (MEM) seeds and super MEM seeds. SOAP3-dp is an aligner based on graphics processing unit (GPU) computing and adopts a similar seeding approach to Bowtie2, while GEM adopts a filtration-based approximate string matching approach to extract relevant candidate matches by suitable pigeonhole-like rules. In addition, Novoalign (http://www.novocraft.com) is a proprietary short-read aligner for fully gapped alignments. However, its method has not been published. Although these aligners can efficiently align the majority of short-reads at high speed, they still have difficulties in correctly aligning all reads, even for simulated ones, to large genomes such as the human genome [11] [16]. Hence, it is of great significance to design new short-read aligners to further improve alignment quality.

In this article, we present CUSHAW3, an open-source sensitive and accurate short-read aligner for both base-space and color-space sequences. In our aligner, we have investigated a hybrid seeding approach to improve alignment quality, which incorporates three different seed types: MEM seeds, exact-match *k*-mer seeds and variable-length seeds derived from local alignments, into the alignment pipeline. Furthermore, three techniques: weighted seed pairing heuristic, paired-end (PE) alignment pair ranking and read mate rescuing, have been proposed to facilitate accurate PE alignment. It needs to be stressed that the concept of hybrid seeding has already been implied in some other implementations for short-read alignment. One example is Stampy [17], an aligner for Illumina sequencing, which first aligns reads with BWA (based on inexact-match seeds) and then processes unmapped reads with another seed-and-extend-based approach using exact-match *k*-mers. Another example is TMAP (https://github.com/iontorrent/TMAP), an aligner for Ion Torrent sequencing, which incorporates the alignment approaches from SSAHA (fixed-length *k*-mer seeds) [18], BWA, BWA-SW and BWA-MEM.

The performance of CUSHAW3 has been assessed by aligning both simulated and real short-reads to the human genome in terms of single-end (SE) and PE alignment. For base-space alignment, our aligner is further compared to Novoalign, CUSHAW2, BWA-MEM, Bowtie2 and GEM. The experimental results reveal that CUSHAW3 is consistently superior to CUSHAW2, BWA-MEM, Bowtie2 and GEM for both SE and PE alignments. Furthermore, our aligner achieves better PE alignment quality than Novoalign for short-reads with higher error rates. As for the speed, CUSHAW3 is inferior to CUSHAW2, BWA-MEM, Bowtie2 and BWA-MEM, but nearly always faster than Novoalign. As for color-space alignment, our aligner is consistently one of the best aligners in terms of alignment quality at superior speed, compared to SHRiMP2 [19] and BFAST.

## Results

### Evaluation on Base-space Reads

We have evaluated the performance of CUSHAW3 (v3.0.2) by aligning both simulated and real short-reads to the human genome (hg19). This performance is further compared to that of CUSHAW2 (v2.1.10), Novoalign (v3.00.04), BWA-MEM (v0.7.3a), Bowtie2 (v2.1.0) and GEM (v 1.376). All tests are conducted in a workstation with a dual hex-core Intel Xeon X5650 2.67 GHz CPUs and 96 GB RAM, running Linux (Ubuntu 12.04 LTS).

To measure alignment quality, we have used the sensitivity metric, which is calculated by dividing the number of aligned reads by the total number of reads, for both simulated and real reads. For simulated reads, as the true mapping positions are known beforehand, we have further used the recall metric, which is defined as dividing the number of correctly aligned reads by the total number of reads. For simulated reads, an alignment is deemed to be correct if the mapping position has a distance of ≤10 to the true position on the genome. Considering that GEM reports all detected alignments and BWA-MEM might produce multiple primary alignments for a read, we define that a read is deemed to be correctly aligned if any of its reported alignments is correct. To provide fair comparisons, we have configured CUSHAW3, CUSHAW2 and Bowtie2 to report a maximum of 10 alignments for each read and Novoalign to report all repetitive alignments. Detailed alignment parameters of all evaluated aligners can be obtained from Tables S1, S2, S3, S4 and S5 in File S1. In addition, all best values in the following tables have been highlighted in bold.

#### On simulated data

We have simulated three Illumina-like PE datasets from the human genome (hg19) using the wgsim simulator in SAMtools v0.1.18 [20]. All datasets have the same read lengths of 100, but with different mean base error rates: 2%, 4% and 6%. Each dataset comprises one million read pairs with insert-sizes drawn from a normal distribution N(500, 50).

Firstly, we have compared the alignment quality of all evaluated aligners by considering all reported alignments (see Table 1), by setting the minimum mapping quality score (MAPQ) to 0. For the SE alignment, Novoalign yields the best sensitivity and recall for each dataset. CUSHAW3 holds equally best sensitivity for the dataset with 2% error rate, and is consistently the second best for all other datasets. With the increase of error rates, each aligner has experienced some performance drops in terms of both measures. Novoalign has the smallest sensitivity (recall) decrease by 0.02% (2.95%), whereas Bowtie2 shows the most significant sensitivity (recall) decrease by 18.10% (21.66%). CUSHAW3 gives the second smallest performance drop with a sensitivity (recall) decrease by 0.74% (3.76%). With PE information, each aligner gets the alignment quality improved over the SE alignment in terms of both measures. In terms of sensitivity, CUSHAW3, Novoalign and BWA-MEM are consistently the top three aligners for all datasets, and Bowtie2 is the worst. In terms of recall, CUSHAW3 is superior to other aligners for the dataset with 6% error rate, while Novoalign performs best for all remaining datasets. CUSHAW3 outperforms CUSHAW2, BWA-MEM, Bowtie2 and GEM for each dataset. Similar to the SE alignment, the error rates also have significant impact on the sensitivity and recall for each aligner. As the error rate grows, Novoalign gives the least significant performance drop and CUSHAW3 the second least in terms of sensitivity. However, in terms of recall, CUSHAW3 has the smallest performance decrease.

**Table 1:** Alignment quality on simulated reads (in %).

Aligner	2%			4%			6%		
	Sensitivity	Recall*	Recall**	Sensitivity	Recall*	Recall**	Sensitivity	Recall*	Recall**
SE									
CUSHAW3	**100.00**	99.04	95.96	99.92	97.85	94.81	99.26	95.28	92.32
CUSHAW2	99.95	99.00	95.96	99.33	97.61	94.64	95.45	92.84	90.04
Novoalign	**100.00**	**99.59**	**96.20**	**99.97**	**98.81**	**95.42**	**99.98**	**96.65**	**93.33**
BWA-MEM	99.99	95.95	95.95	99.59	94.33	94.33	97.38	89.86	89.86
Bowtie2	99.30	95.69	92.98	93.64	87.59	85.20	81.20	74.03	72.03
GEM	99.76	99.02	95.46	97.08	92.28	89.09	90.46	77.64	75.11
PE									
CUSHAW3	**100.00**	99.54	97.35	**100.00**	99.14	96.99	99.96	**98.06**	**96.28**
CUSHAW2	99.73	99.43	97.27	99.36	98.71	96.61	96.47	95.07	93.16
Novoalign	**100.00**	**99.87**	97.57	**100.00**	**99.23**	96.93	**100.00**	97.13	94.88
BWA-MEM	**100.00**	97.59	**97.59**	**100.00**	97.11	**97.11**	99.88	95.55	95.55
Bowtie2	99.45	98.53	96.41	93.54	91.52	89.54	80.29	77.37	75.68
GEM	**100.00**	99.20	96.85	99.79	98.06	95.77	97.99	93.24	91.15

* means the recall is calculated from all reported alignments per read and ** means the recall is calculated form the first alignment occurrence per read.

Secondly, we have further assessed all evaluated aligners by only considering the first alignment occurrence per read in the SAM file, with the minimum MAPQ set to 0. This alignment sampling does not affect the sensitivity of each aligner, but might change the recall. Hence, all aligners are only compared in terms of recall in this evaluation (see Table 1). In terms of SE alignment, Novoalign achieves the best recall for each dataset and CUSHAW3 performs second best. In terms of PE alignment, CUSHAW3 is superior to all other aligners for the dataset with 6% error rate, while BWA-MEM performs best for the remaining datasets. Bowtie2 is the worst for each case. Some readers may argue that for a read with multiple alignments, we can choose the alignment with the largest MAPQ instead of the first alignment. Actually, we can explain that for each evaluated aligner, our evaluation by choosing the first alignment occurrence per read is consistent with that by selecting the alignment with the largest MAPQ from amongst the multiple alignments. Firstly, GEM does not compute MAPQs, but stratifies all identified alignments of a read in ascending order of string distance (Hamming distance or edit distance) [12]. This suggests that GEM implicitly considers the first alignment occurrence as the best candidate in terms of the specified distance metric. Secondly, when enabling multiple alignments per read (by option “-k”), Bowtie2 assigns a pseudo MAPQ to the identified alignments of a read and then reports them in descending order of alignment score (see Bowtie2 manual and command-line help). Thirdly, CUSHAW3, CUSHAW2 and BWA-MEM rank the alignments and build a sorted list with the alignments ordered from best to worst. Both CUSHAW3 and CUSHAW2 produce the same MAPQ for the alignments (possibly with slight differences depending on the degree of soft clipping). BWA-MEM computes one MAPQ for one alignment, but ensures that the MAPQ of each alignment must not exceed that of the best alignment, i.e. the first alignment in the sorted list (refer to the source code). Finally, for a read with multiple alignments, Novoalign first ranks the multiple alignments and then determines the significance of the alignments based on the alignment score difference between the best alignment and the rest of the alignments (see the Novoalign manual). Since the source code of Novoalign is closed, we are not able to reveal more details about the mapping quality score computation and alignment reporting. However, after having examined the alignments on the simulated data, we found that the first alignment occurrences hold the largest MAPQs for each dataset.

**Figure 1:**
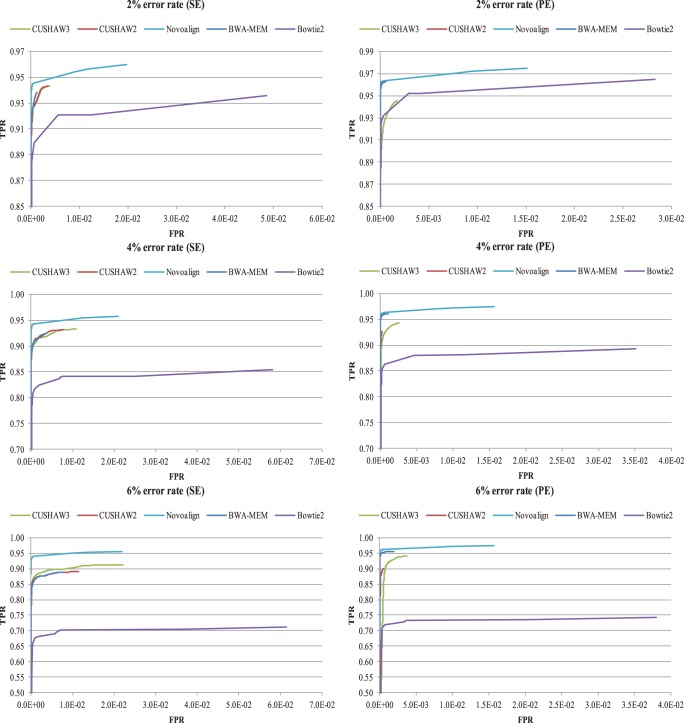
ROC curves of all evaluated aligners on the simulated data with the minimum MAQP>0.

Thirdly, we have generated the receiver operating characteristic (ROC) curves by plotting the true positive rate (TPR) against the false positive rate (FPR) in terms of MAPQ, where for each dataset all alignments are sorted in descending order of MAPQ. For any MAPQ *q*, we compute TPR by dividing the number of correctly aligned reads, whose MAPQs are not less than *q*, by the total number of reads, and FPR by dividing the number of incorrectly aligned reads, whose MAPQs are not less than *q*, by the number of aligned reads whose MAPQs are also not less than *q*. In this evaluation, we have merely taken into account the alignments whose MAPQs are greater than 0. As GEM does not compute MAPQs, it has been excluded. For Bowtie2, we have disabled the option “-k” to enable meaningful MAPQ and have used the default setting to report at most one alignment per read. CUSHAW2 and CUSHAW3 have both been configured to report at most one alignment per read for the SE and PE alignments. For Novoalign, we have used the “-r Random” option to report at most one alignment for a single read. Figure 1 shows the ROC curves for all evaluated aligners on the simulated data. We can see that Novoalign produces the most significant MAPQs for each case.

#### On real data

Finally, we have assessed all aligners using three real PE datasets produced from the Illumina sequencing. All datasets are publicly available and named after their accession numbers in the NCBI sequence read archive (see Table 2). The performance of each aligner is evaluated from two aspects: one is to calculate the sensitivity from all reported alignments; and the other is to calculate the sensitivity after removing the alignments with low aligned base proportion per read. This is because we have observed that some alignments, produced by Novoalign, BWA-MEM, Bowtie2 and GEM, have low aligned base proportion per read (typically <50%) due to soft clipping. Intuitively, such short alignments to the genome are supposed to have higher probabilities to be false positives compared to those aligned to the reference in full lengths (or with high aligned base proportion per read). However, this is not surly the case, especially when there are large indels at the end of the read. In such cases, the correct alignments of the read may be shortened with soft-clipping. However, it is still of great significance to re-evaluate the sensitivity of each aligner by removing the alignments with <50% aligned base proportion per read, which may more truly reflect the alignment quality of an aligner on real data.

**Table 2:** Real dataset information.

Name	Type	Length	No. of Reads	Mean Insert
SRR034939	PE	100	36,201,642	525*
SRR211279	PE	100	50,937,050	302*
ERR024139	PE	100	53,653,010	313*

* estimated using CUSHAW3.

Table 3 shows the alignment quality of all evaluated aligners with or without alignment removal, where the minimum MAPQ threshold is set to 0. For each value *x*/*y* in the table, *x* is the sensitivity calculated from all reported alignments and *y* is the sensitivity after removing the alignments with <50% aligned base proportion per read. Without alignment removal, in terms of SE alignment, CUSHAW3 aligned the most reads for each dataset and GEM is the worst. In terms of PE alignment, BWA-MEM gives the best sensitivity and CUSHAW3 is the second best for all datasets. However, after alignment removal, the sensitivities of both BWA-MEM and Novoalign significantly drop for all datasets in terms of both SE and PE alignment. Bowtie2 keeps its SE sensitivity, but has a slight decrease in PE sensitivity. GEM has also experienced significant PE sensitivity drops for all datasets. Both CUSHAW2 and CUSHAW3 keep their sensitivities unchanged for each case. With alignment removal, CUSHAW3 is consistently superior to all other aligners for each dataset in terms of both SE and PE alignment. In addition, we have shown how the sensitivity (without alignment removal) varies as MAPQ changes (see Figure S1 in File S1). In this evaluation, all alignments are first sorted in descending order of MAPQs and then the sensitivity corresponding to any MAPQ *q* (0≤*q*≤255) is computed by taking into account all alignments whose MAPQs are not less than *q*.

**Table 3:** Alignment quality on real reads (in %).

Aligner	SRR034939	SRR211279	ERR024139
SE			
CUSHAW3	**98.48/98.48**	**99.25/99.25**	**99.12/99.12**
CUSHAW2	93.86/93.86	96.76/96.76	96.74/96.74
Novoalign	96.80/91.27	98.44/98.28	98.49/97.50
BWA-MEM	98.30/97.14	99.17/98.58	99.07/98.50
Bowtie2	95.56/95.56	97.13/97.13	97.20/97.20
GEM	93.69/93.69	95.10/95.10	94.82/94.81
PE			
CUSHAW3	98.92/**98.92**	99.46/**99.46**	99.33/**99.33**
CUSHAW2	94.38/94.38	96.94/96.94	96.92/96.92
Novoalign	98.00/94.23	99.25/98.85	99.13/97.87
BWA-MEM	**99.06/**97.14	**99.49/**98.58	**99.36/**98.50
Bowtie2	96.23/95.56	97.31/97.13	97.39/97.20
GEM	95.52/93.69	96.16/95.10	96.15/94.81

For each value *x*/*y*, *x* is the sensitivity calculated from all reported alignments and *y* is the sensitivity after removing the alignments with <50% aligned base proportion per read.

#### Speed and memory comparison

Besides alignment quality, the speed of each aligner has been evaluated using the aforementioned simulated and real data. We have run each aligner with 12 threads on the aforementioned workstation. For fair comparisons, GEM has counted in the SAM format conversion time (sometimes takes >50% of the overall runtime), as every other aligner reports alignments in SAM format. In addition, all runtimes are measured in wall clock time.

Table 4 shows the runtime (in minutes) of all evaluated aligners on both simulated and real data. For the simulated data, Novoalign is the slowest for nearly all cases, with an exception that CUSHAW3 performs worst in terms of PE alignment for the dataset with 4% error rate. For the SE alignment, BWA-MEM runs fastest on the datasets with 2% and 4% error rates, while Bowtie2 performs best for the dataset with 6% error rate. For the PE alignment, BWA-MEM is superior to all other aligners for each dataset, with an exception that GEM has a tie with BWA-MEM for the dataset with 4% error rate. In addition, the runtimes of both Novoalign and CUSHAW3 are more sensitive to the error rates compared to other aligners. For the real data, BWA-MEM is consistently the fastest for each case and Novoalign is the worst.

**Table 4:** Runtimes (in minutes) on simulated and real base-space reads.

Simulated	2%		4%		6%	
	SE	PE	SE	PE	SE	PE
CUSHAW3	3.4	6.2	3.7	8.1	3.9	10.7
CUSHAW2	2.5	2.5	2.8	2.9	2.9	3.1
Novoalign	6.7	6.6	38.1	7.0	131.7	12.6
BWA-MEM	**1.4**	**2.3**	**1.9**	**1.9**	2.0	**2.1**
Bowtie2	2.1	3.6	2.0	2.7	**1.7**	2.2
GEM	5.7	2.4	5.9	**1.9**	5.4	2.0
**Real**	**SRR034939**		**SRR211279**		**ERR024139**	
	**SE**	**PE**	**SE**	**PE**	**SE**	**PE**
CUSHAW3	62.0	292.4	78.6	317.9	85.1	264.1
CUSHAW2	38.0	38.5	47.2	49.0	51.4	50.5
Novoalign	862.1	497.6	2,024.0	1,243.8	754.2	460.3
BWA-MEM	**25.2**	**25.9**	**24.6**	**26.1**	**27.7**	**30.9**
Bowtie2	50.4	55.9	79.1	69.5	78.0	72.7
GEM	53.0	34.4	72.2	44.7	68.3	51.0

**Figure 2:**
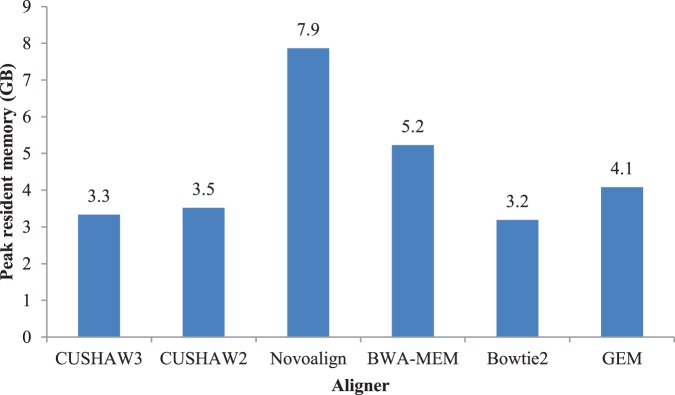
Peak resident memory of all evaluated aligners.

As for memory consumption, the peak resident memory of each aligner has been calculated by performing PE alignment on the dataset with 2% error rate using a single CPU thread (see Figure 2). Bowtie2 takes the least memory of 3.2 GB and Novoalign consumes the most memory of 7.9 GB. CUSHAW3 and CUSHAW2 have a memory footprint of 3.3 GB and 3.5 GB, respectively. For BWA-MEM and GEM, the peak resident memory is 5.2 GB and 4.1 GB, respectively.

### Evaluation on Color-space Reads

In addition to base-space alignment, we have evaluated the performance of CUSHAW3 for color-space alignment, and have further compared our aligner to SHRiMP2 (v2.2.3) and BFAST (v0.7.0a). In this evaluation, we have simulated two mate-paired datasets (read lengths are 50 and 75) from the human genome using the ART (v1.0.1) simulator [21]. Each dataset has 10% coverage of the human genome (resulting in 6,274,322 reads in the 50-bp dataset and 4,182,886 reads in the 75-bp dataset) and has an insert-size 200±20.

Both CUSHAW3 and SHRiMP2 are configured to report up to 10 alignments per read and BFAST to report all alignments with the best score for each read. Each aligner conducts mate-paired alignments and runs with 12 threads on the aforementioned workstation. Table 5 shows the alignment quality and the runtimes of the three aligners. In terms of sensitivity, CUSHAW3 outperforms both SHRiMP2 and BFAST for the 50-bp dataset, while BFAST is the best for the 75-bp dataset. When considering all reported alignments, SHRiMP2 produces the best recall and CUSHAW3 performs second best for every dataset. When only considering the first alignment occurrence per read, CUSHAW3 is superior to both SHRiMP2 and BFAST for each dataset. In terms of speed, CUSHAW3 is the fastest for each case. On average, CUSHAW3 achieves a speedup of 9.5 (and 11.9) over SHRiMP2 (and BFAST). In particular, for the 75-bp dataset, our aligner runs 13.5× and 19.9× faster than SHRiMP2 and BFAST, respectively. In addition, for each aligner, the recall gets improved as the read length increases.

**Table 5:** Alignment quality and runtimes on color-space reads.

Dataset	Measure	CUSHAW3	SHRiMP2	BFAST
50-bp	Sensitivity	**92.13**	91.55	88.94
	Recall*	86.28	**88.58**	81.01
	Recall**	**84.72**	84.22	81.01
	Time(min)	**41**	227	160
75-bp	Sensitivity	92.27	92.33	**93.44**
	Recall*	91.16	**91.24**	86.14
	Recall**	**89.31**	88.15	86.14
	Time(min)	**20**	263	389

Same as Table 1.

### Evaluation on GCAT Benchmarks

Finally, we have evaluated the performance of our aligner using the public benchmarks at GCAT (http://www.bioplanet.com/gcat), which is a free collaborative platform for comparing multiple genome analysis tools across a standard set of metrics. In this evaluation, we have compared CUSHAW3 to CUSHAW2, BWA-MEM and Novoalign in terms of alignment quality and variant calling. The evaluation results for each aligner can also be obtained from our CUSHAW3 homepage (http://cushaw3.sourceforge.net).

In terms of alignment quality, two Illumina-like SE datasets as well as two Illumina-like PE datasets have been used. For the two datasets of each alignment type, one has small indels in reads (the small-indel dataset) and the other contains large indels (the large-indel dataset). All of the four datasets are simulated from the human genome and have read length 100, where there are 11,945,249 reads in each SE dataset and 11,945,250 reads in each PE dataset. To be consistent with the GCAT standard evaluations, both CUSHAW2 and CUSHAW3 are configured to report at most one alignment per read for both the SE and PE alignments. Table 6 shows the alignment results of all evaluated aligners. In terms of SE alignment, CUSHAW3 yields the best sensitivity for both datasets. The best recall is achieved by CUSHAW3, CUSHAW2 and BWA-MEM on the small-indel dataset and by BWA-MEM on the large-indel dataset. CUSHAW3 performs better than Novoalign for each case. CUSHAW2 outperforms Novoalign on the small-indel dataset in terms of both sensitivity and recall, while yielding smaller recall on the large-indel dataset. In terms of PE alignment, BWA-MEM performs best for each case and CUSHAW3 is the second best. On the small-indel dataset, CUSHAW2 outperforms Novoalign in terms of both sensitivity and recall. On the large-indel dataset, CUSHAW2 yields better sensitivity than Novoalign, while Novoalign gives better recall. In addition, the alignment accuracy of different aligners has been further compared by plotting the percentage of incorrectly aligned reads against the percentage of correctly aligned reads with respect to MAPQs. In this plotting, all alignments of each aligner are first sorted in descending order of MAPQ and then the correct and incorrect percentages are calculated. Figures S2, S3, S4 and S5 in File S1 show the alignment accuracy comparison for both SE and PE alignments. In terms of SE and PE alignment, Novoalign is superior to all other aligners and BWA-MEM is the second best with respect to the plotting. CUSHAW2 and CUSHAW3 have demonstrated nearly identical curves, and still need further improvement on the calculation of MAPQs compared to Novoalign and BWA-MEM.

**Table 6:** Alignment results on GCAT benchmarks.

Dataset	Measure	CUSHAW3	CUSHAW2	Novoalign	BWA-MEM
SE					
Small indels	Sensitivity	**100.00**	99.86	97.56	99.99
	Recall	**97.52**	**97.52**	97.47	**97.52**
Large indels	Sensitivity	**100.00**	99.50	97.56	99.99
	Recall	97.37	97.04	97.35	**97.40**
PE					
Small indels	Sensitivity	**100.00**	99.99	98.85	**100.00**
	Recall	99.06	99.05	98.83	**99.22**
Large indels	Sensitivity	**100.00**	99.71	98.84	**100.00**
	Recall	98.91	98.62	98.69	**99.08**

In terms of variant calling, a real exome sequencing dataset has been used in this test. This dataset is comprised of Illumina 100-bp PE reads and has 30× coverage of the human exome. In this test, we have used SAMtools as the variant caller. Table 7 shows the variant calling results, where the novel single nucleotide polymorphisms (SNPs) in the dbSNP database are not taken into account. BWA-MEM yields the maximum sensitivity and Novoalign performs second best. In terms of specificity, Novoalign achieves the best performance, while CUSHAW2 and CUSHAW3 tie for the second place. As for Ti/Tv ratio, CUSHAW2 produces the maximum value of 2.323 and Novoalign gives the second best value of 2.289. CUSHAW3 and BWA-MEM are joint third. BWA-MEM identifies the most correct SNPs, while Novoalign yields the most correct indels. Compared to CUSHAW2, CUSHAW3 holds a smaller Ti/Tv ratio, but has an improved sensitivity as well as identifies more correct SNPs and indels. In addition, we have given a Venn diagram (see Figure S6 in File S1) to show the variant concordance between the evaluated aligners.

**Table 7:** Variant calling results on a GCAT benchmark.

Aligner	Sensitivity	Specificity	Ti/Tv	CorrectSNP	Correct Indel
CUSHAW3	83.74	99.9930	2.285	115,709	5,974
CUSHAW2	83.51	99.9930	**2.323**	112,727	5,841
Novoalign	84.10	**99.9951**	2.289	121,992	**9,416**
BWA-MEM	**85.30**	99.9926	2.285	**124,459**	9,232

Sensitivity = TP/(TP+FN), specificity = TN/(TN+FP) and Ti/Tv is the ratio of transitions to transversions in SNPs.

## Discussion

In this article, we have presented CUSHAW3, an open-source tool for sensitive and accurate short-read alignment to large genomes, such as the human genome. This aligner is designed based on the well-known seed-and-extend heuristic and has introduced a hybrid seeding approach to improve alignment quality for both SE and PE alignments. This hybrid seeding approach works by incorporating three different seed types, namely MEM seeds, exact-match *k*-mer seeds and variable-length seeds derived from local alignments, into our alignment pipelines. Furthermore, we have proposed three critical bioinformatics techniques: weighted seed-paring heuristic, PE alignment pair ranking and read mate rescuing, to facilitate accurate PE alignments. CUSHAW3 accepts short-reads represented in FASTA, FASTQ, SAM/BAM [20] format, which can be uncompressed or zlib-compressed, and provides an easy-to-use and well-structured interface as well as a more detailed documentation about the installation and usage. In addition, our aligner produces PHRED [22] compliant MAPQs for all alignments and reports them in SAM format. This enables seamless integration of our aligner with established downstream analysis tools like SAMtools [20] and GATK [23].

CUSHAW3 provides support for both base-space and color-space alignments. For base-space alignment, we have assessed the performance of CUSHAW3 and other top-performing short-read aligners: Novoalign, CUSHAW2, BWA-MEM, Bowtie2 and GEM using simulated as well as real reads from the human genome. For both simulated and real data, we have employed the sensitivity measure. Additionally, the recall measure has been further used on simulated data, as the ground truth of alignments is known beforehand. On simulated data, CUSHAW3 achieves consistently better alignment quality (by considering all reported alignments) than CUSHAW2, BWA-MEM, Bowtie2 and GEM in terms of both SE and PE alignment. Compared to Novoalign, CUSHAW3 has comparable PE alignment performance for short-reads with low error rates, but performs better for short-reads with high error rates. On real data, CUSHAW3 achieves the highest SE and PE sensitivities for each dataset. As for speed, CUSHAW3 does not have any advantage over CUSHAW2, BWA-MEM, Bowtie2 and GEM, but shows to be nearly always faster than Novoalign. In terms of color-space alignment, we have evaluated and compared the performance of CUSHAW3, SHRiMP2 and BFAST using simulated mate-paired color-space reads. The results show that CUSHAW3 is consistently one of the best color-space aligners in terms of alignment quality. Moreover, on average CUSHAW3 is one order-of-magnitude faster than both SHRiMP2 and BFAST on the same hardware configurations. From our evaluations, we have observed that a considerable number of alignments, reported by Novoalign, BWA-MEM, Bowtie2 and GEM, have low aligned base proportion per read (<50%), especially for the PE alignments. Furthermore, even though both CUSHAW3 and Novoalign are shown to have higher alignment accuracy, some simulated reads still missed their correct alignments. Moreover, this situation becomes even worse as the error rate grows larger. Hence, more research efforts are still required in order to better align short-reads with high error rates. Finally, as shown in our evaluations, the hybrid seeding approach does improve accuracy, but at the expense of speed. To significantly reduce the runtime, one promising solution is the use of GPU computing, as some pioneer work (e.g. [6] [7] [14]) has shown that short-read alignment can significantly benefit from the GPU computing with respect to speed. This acceleration based on special hardware can be considered as part of our future work.

## Methods

### Hybrid Seeding

**Figure 3:**
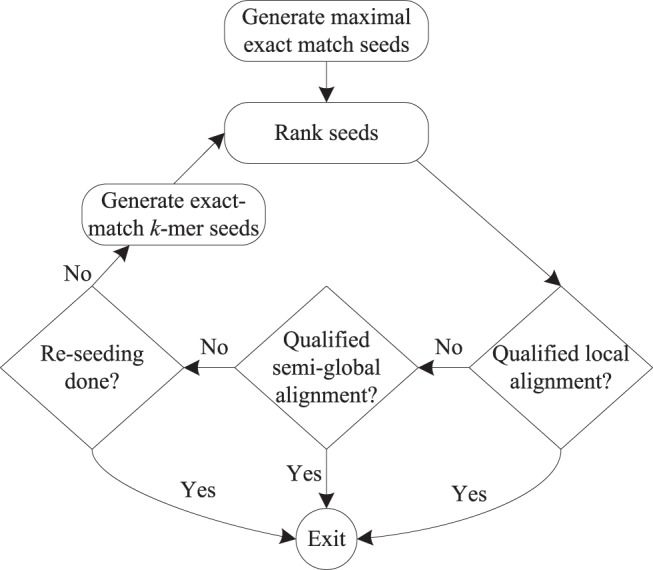
Program workflow of the single-end alignment using hybrid seeding.

Our hybrid seeding approach incorporates MEM seeds, exact-match *k*-mer seeds, and variable-length seeds at different phases of the alignment pipeline. For a single read, the alignment pipeline generally works as follows (see Figure 3).

First, we produce the MEM seeds for both strands of the read based on Burrows-wheeler transform [24] and FM-index [25]. Secondly, from each seed we determine on the genome a potential mapping region for the read, and then perform the Smith-Waterman algorithm [26] to gain the optimal local alignment score between the read and the mapping region. All seeds are subsequently ranked in terms of optimal local alignment score, where greater scores mean higher ranks.

Thirdly, dynamic programing is employed to identify the optimal local alignment of the read to the genome from the highest-ranked seeds. If satisfying the local-alignment constraints, including minimal percentage identity (default = 90%) and aligned base proportion per read (default = 80%), the optimal local alignment will be considered as qualified. Otherwise, we will attempt to rescue the read using a semi-global alignment approach. As an optimal local alignment usually indicates the most similar region on the genome, our semi-global alignment approach takes the optimal local alignment as a variable-length seed, re-computes a new mapping region on the genome and then performs semi-global alignment between the read and the new mapping region to obtain an optimal semi-global alignment. If the optimal semi-global alignment satisfies the global-alignment constraints, including minimal percentage identity (default = 65%) and aligned base proportion per read (default = 80%), this alignment will be deemed to be qualified. This double-alignment approach enables us to capture the alignments with more continuous mismatches and longer gaps. This is because we might fail to get good enough optimal local alignments in such cases, as the positive score for a match is usually smaller than the penalty charged for mismatches and indels.

Finally, when we still fail to get any qualified alignment, this likely means that the true alignment is implied by none of the evaluated MEM seeds. In this case, we attempt to rescue the alignment by re-seeding the read using exact-match *k*-mer seeds. To improve speed, we search all non-overlapping *k*-mers of the read against the genome to identify seed matches. Subsequently, we employ the *k*-mer seeds to repeat the aforementioned alignment process to rescue the read. If we still fail to gain a qualified alignment, we will stop the alignment process and then report this read as unaligned.

### Paired-end Mapping

In comparison with SE alignment, the long-range positional information contained in PE reads usually allow for more accurate short-read alignment, by either disambiguating alignments when one of the two ends aligns to repetitive regions or rescuing one end from its aligned mate. In addition, for aligners based on the seed-and-extend heuristic, the PE information, such as alignment orientations and insert-size of both ends, can aid to significantly reduce the number of noisy seeds prior to the time-consuming alignment extensions. This filtration can be realized through a seed-paring heuristic [11], as a seed determines the alignment orientation of a read and the mapping distance constraint on seed pairs can be inferred from the insert-size of read pairs.

**Figure 4:**
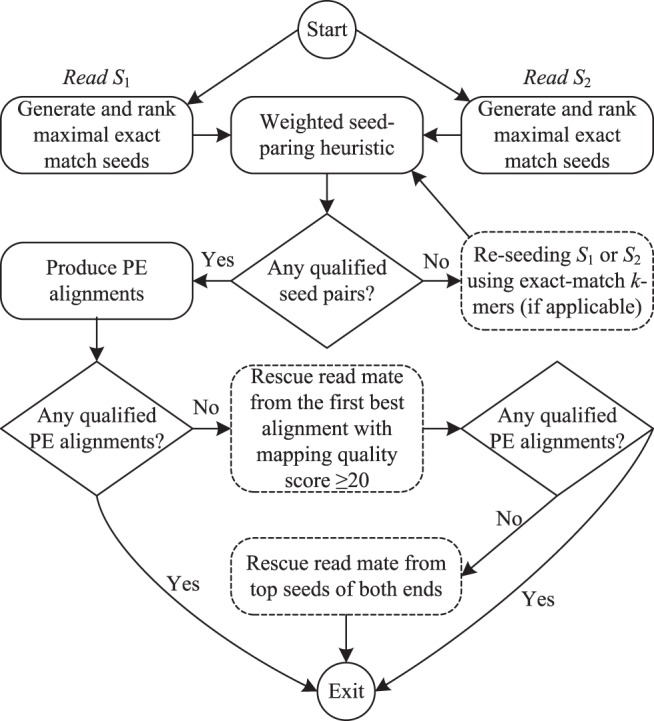
Program workflow of the paired-end alignment with hybrid seeding.

For a read pair *S*_1_ and *S*_2_, our PE alignment pipeline generally works as follows (see Figure 4). First, we generate and rank the MEM seeds, following the same procedure as in SE alignment. Secondly, a weighted seed-paring heuristic is introduced to pair seeds, where only high-quality seeds, whose scores are not less than a minimal score threshold (default = 30), will be taken into account. This heuristic enumerates each high-quality seed pair of *S*_1_ and *S*_2_ to identify all qualified seed pairs that meet the alignment orientation and insert-size requirements. To distinguish all qualified seed pairs in terms of quality, we have calculated a weight for each qualified seed pair and further ranked all of them by a max-heap data structure. This quality-aware feature allows for us to visit all qualified seed pairs in the descending order of quality. Thirdly, if failed to find any qualified seed pair, we will resort to the re-seeding based on exact-match *k*-mers by sequentially checking both ends to see if either of them has not yet been re-seeded. If so, the *k*-mer seeds will be produced for that end and all new seeds will be ranked in the same way as for MEM seeds. Subsequently, we merge all high-quality *k*-mer seeds with the high-quality MEM seeds, and then re-rank all seeds. The seed merge is used because some significant alignments, which are not covered by MEM seeds, may be reflected by *k*-mer seeds, and vice versa. After getting the new list of seeds, we repeat the weighted seed-paring heuristic to gain qualified seed pairs. The seed-paring and re-seeding process will be repetitively continued until either both ends have been re-seeded or any qualified seed pair has been identified. Fourthly, we compute the real alignments of both ends from the qualified seed pairs. An alignment pair will be considered as qualified if their mapping position distance satisfies the insert-size constraint. Similar to the weighted seed-pairing approach, we have also ranked all qualified alignment pairs by means of a max-heap data structure. In this manner, we would expect better alignment pairs to come out earlier in the output. Finally, we attempt to rescue read mates from the best alignments of each end, when failed to pair reads in previous steps.

### Weighted Seed-pairing Heuristic and Alignment Pair Ranking

To guide the production of real PE alignments in a quality-aware manner, we introduce a weighted seed-paring heuristic computing a weight *w* for each qualified seed pair as follows.
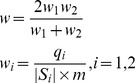
(1)where *q*_i_ is the optimal local alignment score between read *S*_i_ (1≤*i*≤2) and the mapping region derived from the seed, and *m* is the positive score for an alignment match. To rank all qualified seed pairs, we employ *w* as the key of each entry in the max-heap.

In addition to seed pairs, all qualified alignment pairs have been further ranked in terms of weight and edit distance. For an alignment pair, we calculate its weight following Equation (1) with the difference that *q_i_* is not definitely the optimal local alignment score, but might be the optimal semi-global alignment score. This is because an alignment is possibly produced from a semi-global alignment as mentioned above. Furthermore, when two qualified alignment pairs hold the same weights, we further rank them by comparing the sums of the edit distances of each alignment pair. In this case, smaller edit distance sums mean higher ranks.

### Read Mate Rescuing

For unpaired reads, we have employed a read mate rescuing procedure, which attempts to rescue one read from the top hits of its aligned mate by using the paired-end long-range distance information. In general, our rescuing procedure works as follows.

First, the best alignments of the two reads are computed (if available). The read, whose best alignment has a MAPQ exceeding a minimum threshold (default = 20), will be used to rescue its mate. If an optimal alignment satisfying the aforementioned constraints has been gained for the mate, the two reads are considered as paired. Otherwise, we will continue the rescuing process using the alignments with smaller MAPQs. Secondly, if the two reads have not yet been properly paired, we will attempt to pair them from more top hits of both reads. The rescuing process will not stop until the two reads have been properly paired or having reached the maximum number (default = 100) of top seeds for each read. Finally, for unpaired reads, we will report their best alignments (if available) in a SE alignment mode.

This read mate rescuing is usually time-consuming mainly due to two factors. One is the dynamic-programing-based alignment with quadratic time complexity. The other is the maximal insert-size of a read pair, which basically determines the mapping region size of the mate on the genome. In sum, the more reads are paired by seed-pairing heuristic; the less time is taken by the read mate rescuing procedure.

### Color-space Alignment

Most existing color-space aligners encode a nucleotide-based genome as a color sequence and then identify potential short-read alignment hits in color space. However, different approaches may be used to produce the final base-space alignments. For a color-space read, one approach is to identify a final color-space alignment and then convert the color sequence to nucleotides under the guidance of the alignment using dynamic programming [5]. An alternative is to directly perform color-aware dynamic-programming-based alignment by simultaneously aligning all four possible translations [3] [19].

In our aligner, we also convert a nucleotide-based genome to a color sequence and perform short-read alignment in color-space basically following the same workflow as the base-space alignment (mentioned above). For a color-space read, after obtaining a qualified color-space alignment, we must convert the color sequence into a nucleotide sequence. This conversion is accomplished by adopting the dynamic programming approach proposed by Li and Durbin [5]. Subsequently, the translated nucleotide sequence will be re-aligned to the nucleotide-based genome using either local or semi-global alignment depending on how its parent alignment has been produced.

## Supporting Information

File S1 **Supplementary Tables and Figures.**
(PDF)
