# Supplementary material for: CUSHAW3: Sensitive and Accurate Base-Space and Color-Space Short-Read Alignment with Hybrid Seeding
Source: PLoS One. 2014 Jan 22;9(1):e86869. doi: 10.1371/journal.pone.0086869 (PMC3899341; doi:10.1371/journal.pone.0086869)
Supplement: File S1 — Supplementary Tables and Figures. (PDF) [file pone.0086869.s001.pdf]

CUSHAW3 Supplementary Data

Yongchao Liu[1], Bernt Popp[2] and Bertil Schmidt[1]
[1]Institut für Informatik, Johannes Gutenberg Universität Mainz, 55099 Mainz, Germany
[2]Institute of Human Genetics, University of Erlangen-Nuremberg, 91054 Erlangen, Germany

1 Base-space alignment

In our evaluations, we have used the default parameters to build the genome indices for each evaluated aligner. The detailed alignment parameters for both simulated and real data have been given in the following.

1.1 Simulated data

Tables S1 and S2 give the alignment parameters for the single-end and paired-end alignments of simulated data, respectively.

Table S1. Single-end alignment parameters of all evaluated aligners on simulated data

Aligner	Alignment parameters	
CUSHAW3	-t 12 -multi 10	
CUSHAW2	-t 12 -multi 10	
Novoalign	-r All -o SAM -c 12	
BWA-MEM	-t 12	
Bowtie2	-p 12 -k 10 -S	
GEM	gem-mapper	-q 'offset-33' -T 12
	gem-2-sam	-q 'offset-33' --sequence-lengths --expect-single-end-reads

Table S2. Paired-end alignment parameters of all evaluated aligners on simulated data

Aligner	Alignment parameters	
CUSHAW3	-avg_ins 500 -ins_std 50 -t 12 -multi 10	
CUSHAW2	-avg_ins 500 -ins_std 50 -t 12 -multi 10	
Novoalign	-r All -o SAM -i PE 500,50 -c 12	
BWA-MEM	-t 12	
Bowtie2	-X 700 -p 12 -k 10 -S	
GEM	gem-mapper	-q 'offset-33' -p --max-insert-size 700 -T 12
	gem-2-sam	-q 'offset-33' --sequence-lengths --expect-paired-end-reads

1.2 Real data

Tables S3 and S4 give the alignment parameters for the single-end and paired-end alignments of real data, respectively.

Table S3. Single-end alignment parameters of all evaluated aligners on real data

Aligner	Alignment parameters	
CUSHAW3	-t 12 -multi 10	
CUSHAW2	-t 12 -multi 10	
Novoalign	-r All -o SAM -c 12 -k	
BWA-MEM	-t 12	
Bowtie2	-p 12 -k 10 -S	
GEM	gem-mapper	-q 'offset-33' -T 12
	gem-2-sam	-q 'offset-33' --sequence-lengths --expect-single-end-reads

Table S4. Paired-end alignment parameters of all evaluated aligners on real data

Dataset	Aligner	Alignment parameters	
ERR024139	CUSHAW3	-t 12 -multi 10	
	CUSHAW2	-avg_ins 500 -ins_std 0 -t 12 -multi 10	
	Novoalign	-r All -o SAM -i PE 500,0 -c 12 -k	
	BWA-MEM	-t 12	
	Bowtie2	-X 500 -p 12 -k 10 -S	
	GEM	gem-mapper	-q 'offset-33' -p --max-insert-size 500 -T 12
		gem-2-sam	-q 'offset-33' --sequence-lengths --expect-paired-end-reads
SRR211279	CUSHAW3	-t 12 -multi 10	
	CUSHAW2	-avg_ins 500 -ins_std 0 -t 12 -multi 10	
	Novoalign	-r All -o SAM -i PE 500,0 -c 12 -k	
	BWA-MEM	-t 12	
	Bowtie2	-X 500 -p 12 -k 10 –S	
	GEM	gem-mapper	-q 'offset-33' -p --max-insert-size 500 -T 12
		gem-2-sam	-q 'offset-33' --sequence-lengths --expect-paired-end-reads
SRR034939	CUSHAW3	-t 12 -multi 10	
	CUSHAW2	-avg_ins 700 -ins_std 0 -t 12 -multi 10	
	Novoalign	-r All -o SAM -i PE 700,0 -c 12 -k	
	BWA-MEM	-t 12	
	Bowtie2	-X 700 -p 12 -k 10 –S	
	GEM	gem-mapper	-q 'offset-33' -p --max-insert-size 700 -T 12
		gem-2-sam	-q 'offset-33' --sequence-lengths --expect-paired-end-reads

Figure S1 shows the plotting of sensitivity against minimum mapping quality score threshold. In this test, GEM has been excluded because it does not compute mapping quality scores and Bowtie2 has used the default settings to enable the computation of mapping quality scores.

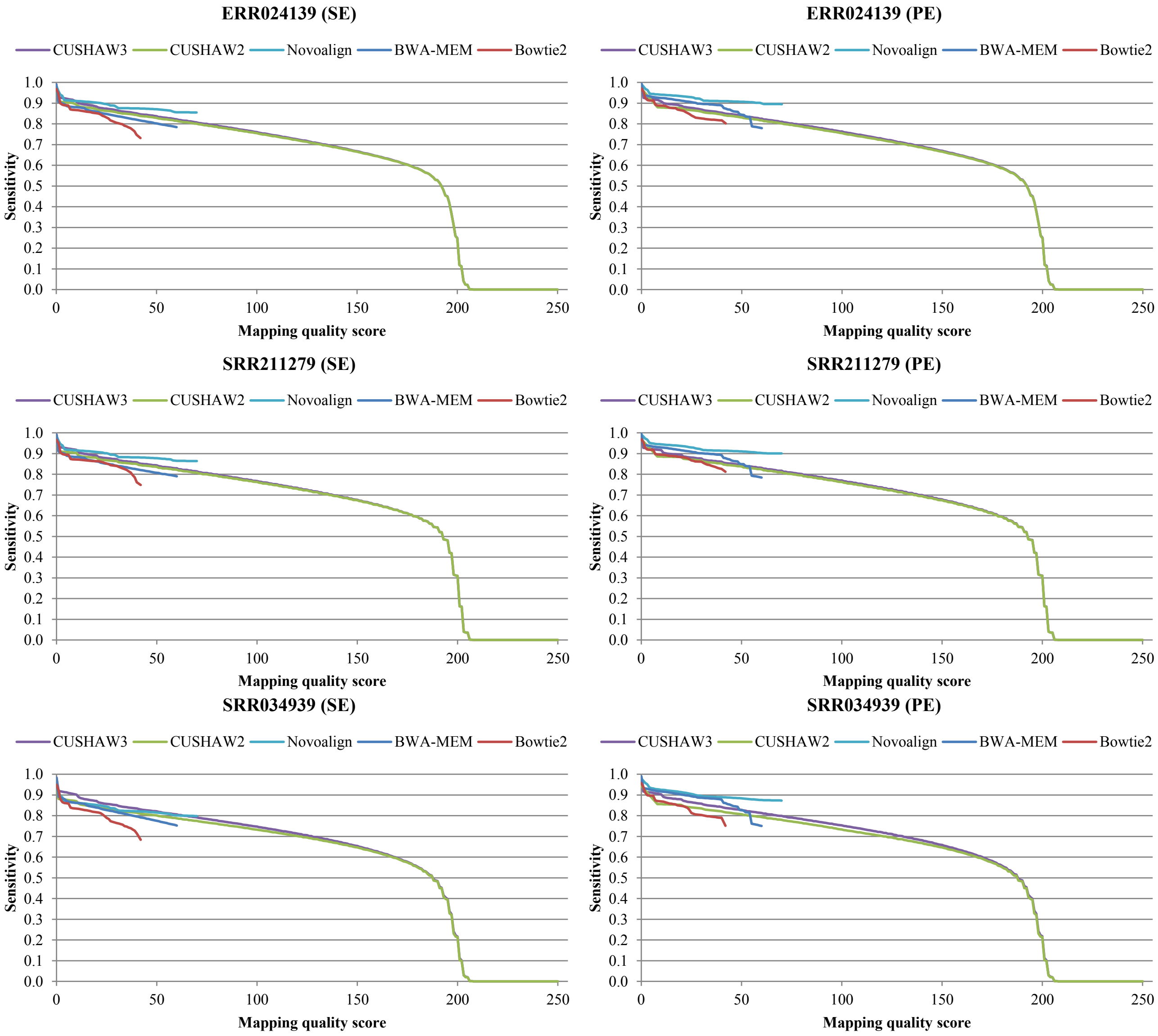

Figure S1 Plotting of sensitivity against mapping quality scores

2 Color-space alignment

CUSHAW3 employs the default parameters to construct color-space genome indices. As the alignment quality of BFAST is sensitive to both the number of genome indices and the spaced-seed masks, we have constructed 10 genome indices using the 10 spaced-seed masks, as recommended in Homer *et al*. (2009), in order to yield high

alignment quality. The following list the command lines for BFAST genome index construction. In addition, Table S5 gives the alignment parameters of all evaluated aligners on both simulated and real datasets.

- bfast index -A 1 -f hg19.fasta -m 111111111111111111 -w 16 -i 1 -n 12
- bfast index -A 1 -f hg19.fasta -m 1111101110111010100101011011111 -w 16 -i 2 -n 12
- bfast index -A 1 -f hg19.fasta -m 10111101011010010110000011010001111111 -w 16 -i 3 -n 12
- bfast index -A 1 -f hg19.fasta -m 10111001101001100100111101010001011111 -w 16 -i 4 -n 12
- bfast index -A 1 -f hg19.fasta -m 11111011011101111011111111 -w 16 -i 5 -n 12
- bfast index -A 1 -f hg19.fasta -m 111111100101001000101111101110111 -w 16 -i 6 -n 12
- bfast index -A 1 -f hg19.fasta -m 11110101110010100010101101010111111 -w 16 -i 7 -n 12
- bfast index -A 1 -f hg19.fasta -m 111101101011011001100000101101001011101 -w 16 -i 8 -n 12
- bfast index -A 1 -f hg19.fasta -m 1111011010001000110101100101100110100111 -w 16 -i 9 -n 12
- bfast index -A 1 -f bfast-hg19.fasta -m 1111010010110110101110010110111011 -w 16 -i 10 -n 12

Table S5. Alignment parameters of all evaluated aligners

Aligner	Parameters	
CUSHAW3	-t 12 -avg_ins 200 -ins_std 20 -multi 10	
SHRiMP2	--no-qv-check -N 12 --insert-size-dist 200,20 --max-alignments 10 -1 filename_F3.fq -2 filename_R3.fq	
BFAST	match	-n 12 -A 1
	localalign	-n 12 -A 1
	postprocess	-n 12 -A 1 -v 200 -s 20 -Y 1

3 Evaluation on GCAT Benchmarks

Figures S2, S3, S4 and S5 show the alignment accuracy comparison by plotting the percentage of incorrectly aligned reads against the percentage of correctly aligned reads with respect to mapping quality scores. Figure S6 shows the variant concordance of all evaluated aligners. All of the figures are automatically generated by the GCAT benchmark website (http://www.bioplanet.com/gcat).

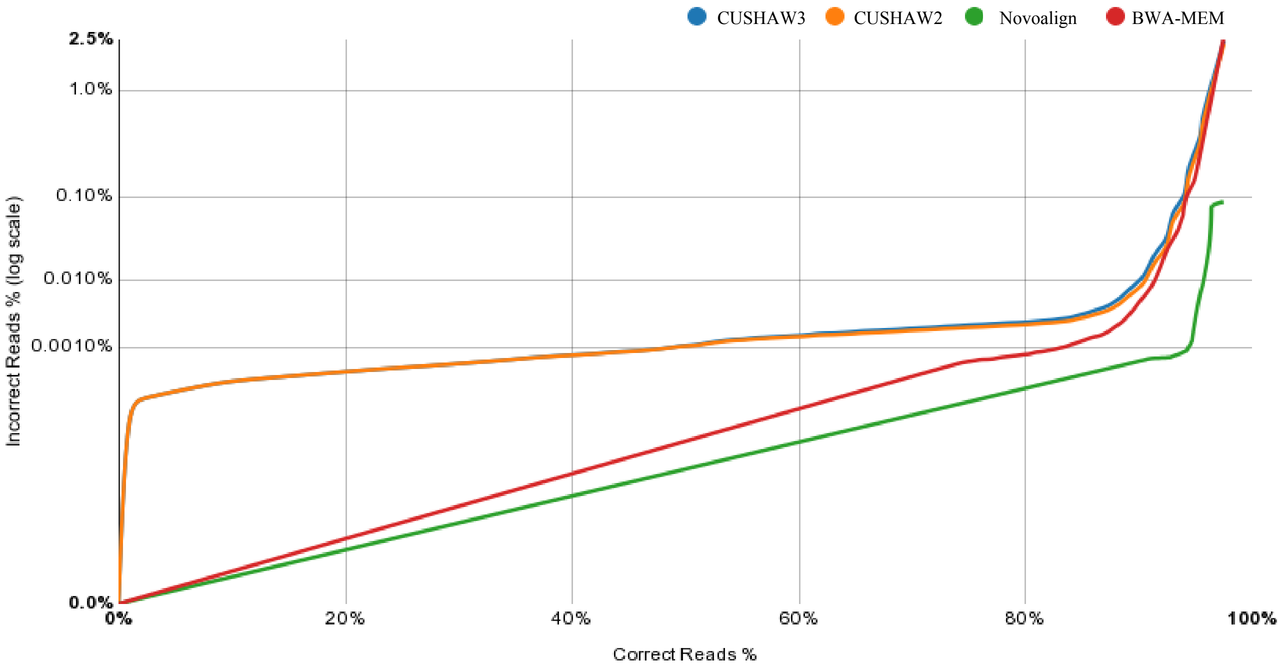

Figure S2. Single-end alignment accuracy on Illumina-like 100-bp single-end reads with small indels

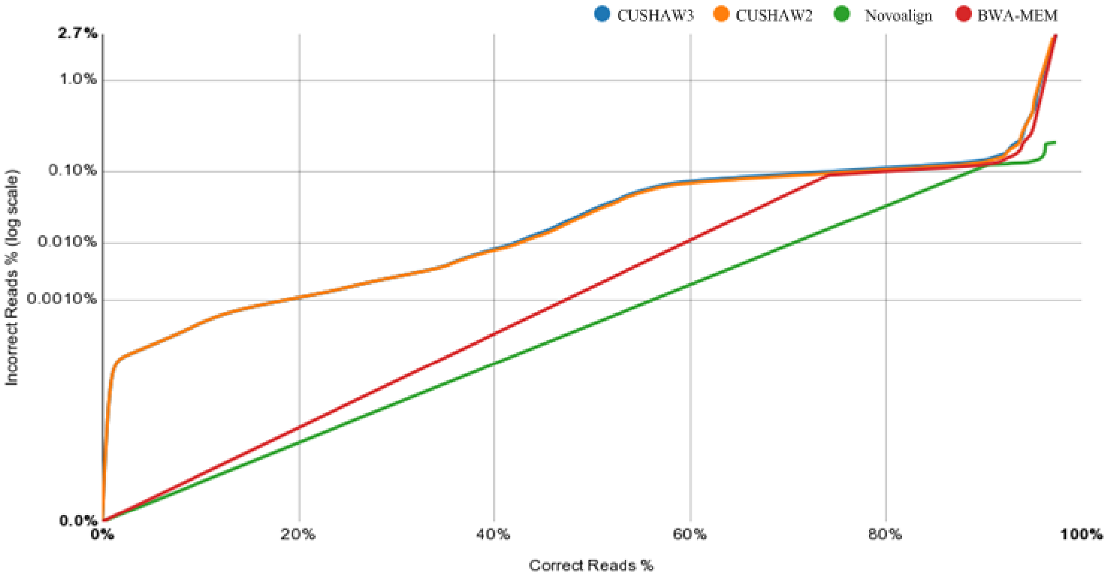

Figure S3. Single-end alignment accuracy on Illumina-like 100-bp single-end reads with large indels

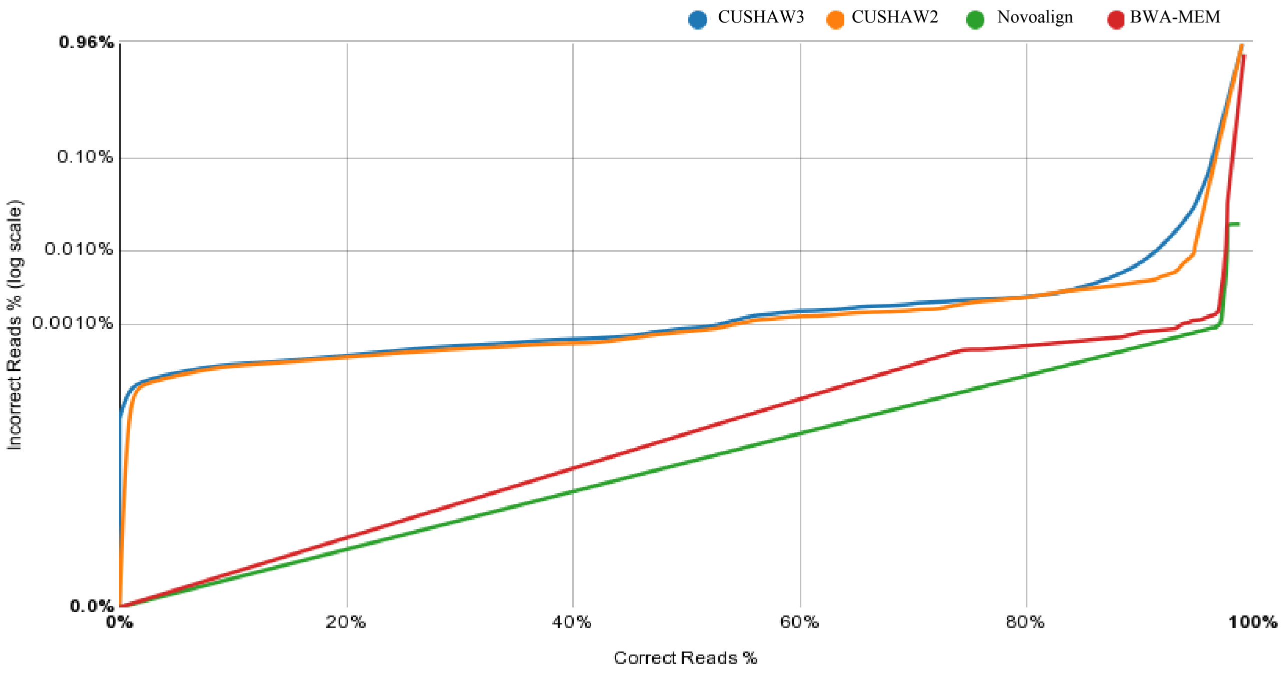

Figure S4. Paired-end alignment accuracy on Illumina-like 100-bp paired-end reads with small indels

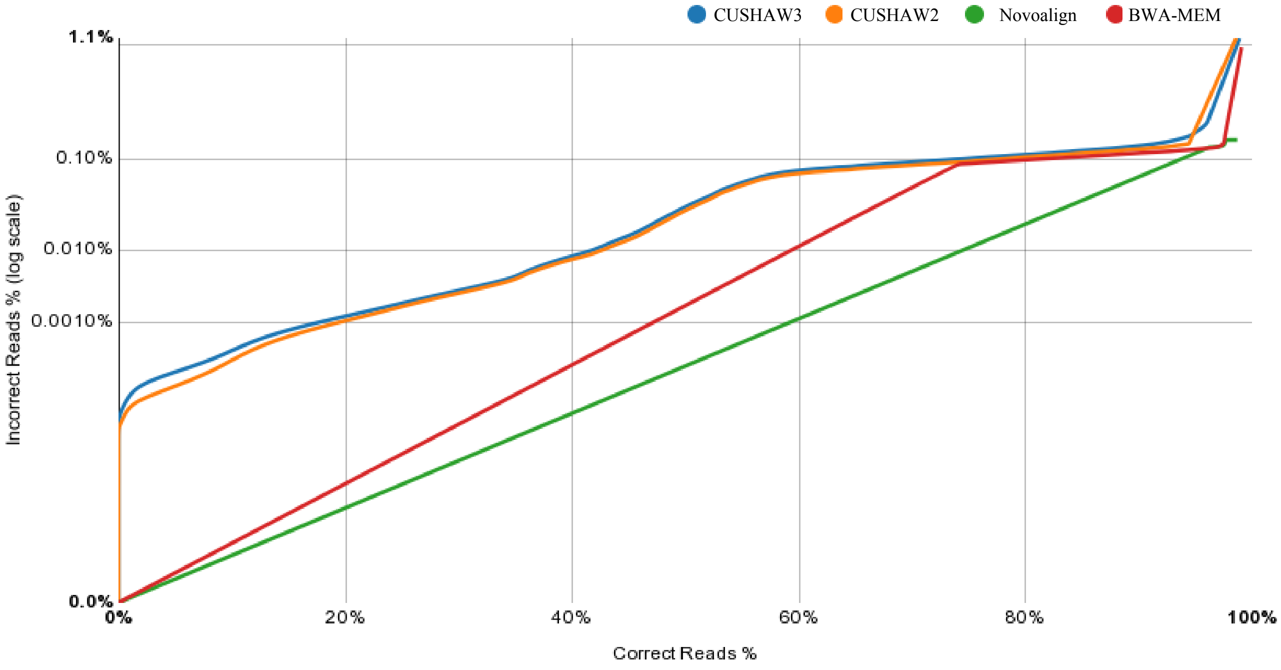

Figure S5. Paired-end alignment accuracy on Illumina-like 100-bp paired-end reads with large indels

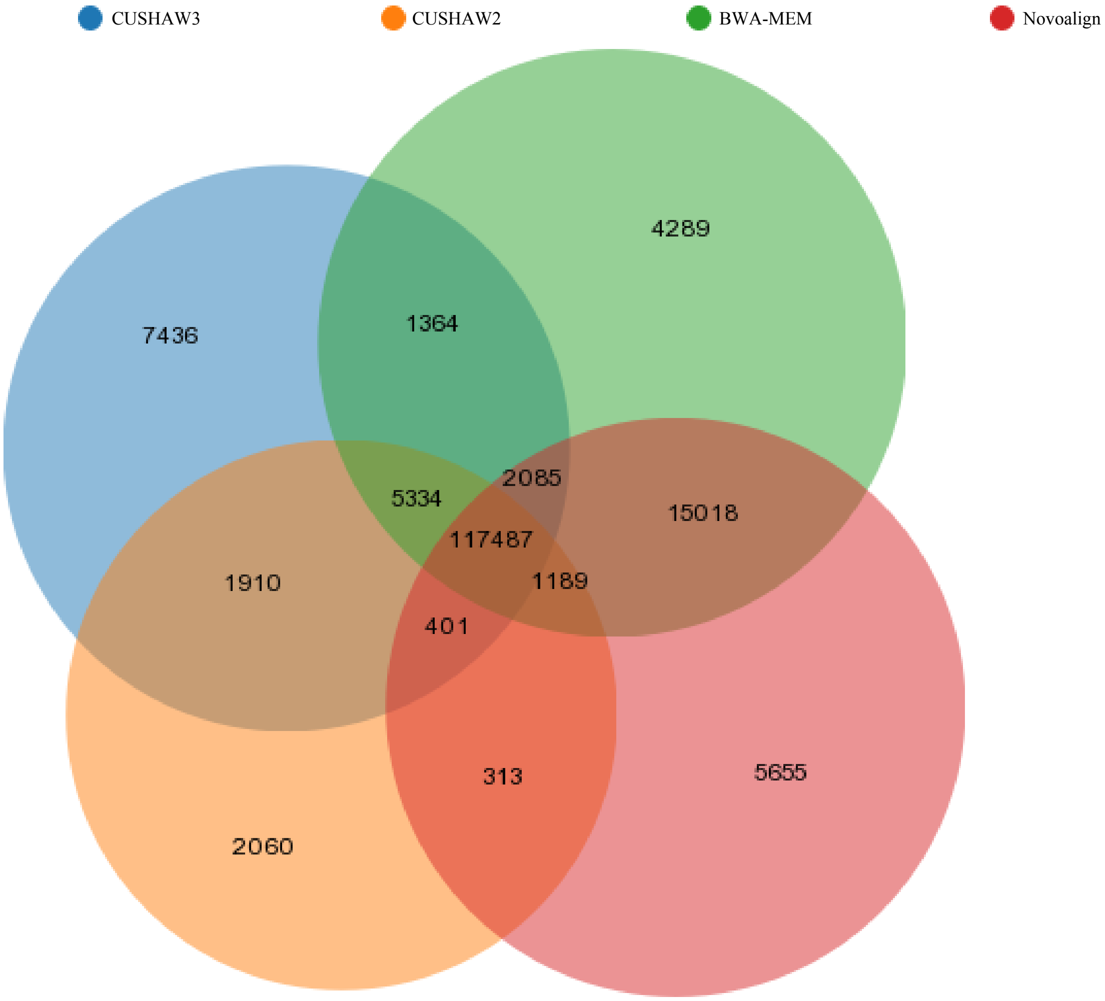

Figure S6. Variant concordance between different aligners on human exome sequencing data

References

Homer,N. *et al*. (2009) BFAST: an alignment tool for large scale genome resequencing. *PLOS one*, 4(11): e7767
